# Maternal Malaria Induces a Procoagulant and Antifibrinolytic State That Is Embryotoxic but Responsive to Anticoagulant Therapy

**DOI:** 10.1371/journal.pone.0031090

**Published:** 2012-02-07

**Authors:** John W. Avery, Geoffrey M. Smith, Simon O. Owino, Demba Sarr, Tamas Nagy, Stephen Mwalimu, James Matthias, Lauren F. Kelly, Jayakumar S. Poovassery, Joab D. Middii, Carlos Abramowsky, Julie M. Moore

**Affiliations:** 1 Department of Infectious Diseases and Center for Tropical and Emerging Global Diseases, University of Georgia, Athens, Georgia, United States of America; 2 Centre for Global Health Research, Kenya Medical Research Institute, Kisumu, Kenya; 3 Department of Veterinary Pathology, University of Georgia, Athens, Georgia, United States of America; 4 Department of Epidemiology and Biostatistics, University of Georgia, Athens, Georgia, United States of America; 5 Department of Pathology and Laboratory Medicine, Emory University, Atlanta, Georgia, United States of America; University of Copenhagen, Denmark

## Abstract

Low birth weight and fetal loss are commonly attributed to malaria in endemic areas, but the cellular and molecular mechanisms that underlie these poor birth outcomes are incompletely understood. Increasing evidence suggests that dysregulated hemostasis is important in malaria pathogenesis, but its role in placental malaria (PM), characterized by intervillous sequestration of *Plasmodium falciparum*, proinflammatory responses, and excessive fibrin deposition is not known. To address this question, markers of coagulation and fibrinolysis were assessed in placentae from malaria-exposed primigravid women. PM was associated with significantly elevated placental monocyte and proinflammatory marker levels, enhanced perivillous fibrin deposition, and increased markers of activated coagulation and suppressed fibrinolysis in placental plasma. Submicroscopic PM was not proinflammatory but tended to be procoagulant and antifibrinolytic. Birth weight trended downward in association with placental parasitemia and high fibrin score. To directly assess the importance of coagulation in malaria-induced compromise of pregnancy, *Plasmodium chabaudi* AS-infected pregnant C57BL/6 mice were treated with the anticoagulant, low molecular weight heparin. Treatment rescued pregnancy at midgestation, with substantially decreased rates of active abortion and reduced placental and embryonic hemorrhage and necrosis relative to untreated animals. Together, the results suggest that dysregulated hemostasis may represent a novel therapeutic target in malaria-compromised pregnancies.

## Introduction

Recent estimates propose that nearly 55 million pregnant women are at high risk for *Plasmodium falciparum* infection annually [1]. Aside from significant maternal morbidity, a critical clinical feature of this infection is low infant birth weight (LBW; <2500 g) secondary to intrauterine growth restriction and/or premature birth [2]. Each year, in Sub-Saharan Africa as many as 363,000 neonates die from malaria-associated LBW [2]. A large proportion of these cases are attributed to malaria-induced maternal anemia and placental, inflammatory pathology and resultant functional insufficiency [2]–[6]. In addition, among pregnant women living in low transmission conditions, who have little pre-existing immunity to malaria, this infection can result in abortion and stillbirth [2].

The major pathological features of malaria during pregnancy that are associated with poor birth outcomes are accumulation of infected red blood cells (iRBCs) in the maternal blood space of the placenta and the subsequent inappropriate maternal inflammatory response to these parasites, a syndrome referred to as placental malaria (PM). Although PM and its consequences for mother and fetus have been well studied, the precise mechanisms of pathology continue to elude investigators. Malarial pathogenesis is commonly attributed to infiltration of immune effector cells and excessive proinflammatory cytokine release in response to sequestered parasites [4], but this proinflammatory immunopathology may not fully account for PM pathogenesis. A universally described histopathological feature of malarious placentae is excessive deposition of fibrin, the end-product of the coagulation cascade [5]. However, an independent role for fibrin in PM-induced adverse birth outcomes has been directly examined in only two studies. Menendez et al. found that malaria-infected placentae with >30% of fetal villi engulfed in fibrin were significantly associated with LBW due to preterm delivery [6]. Additionally, Crocker et al. established an association between placental parasitemia, LBW, and syncytiotrophoblast lesions associated with fibrin-type fibrinoid deposition [3]. In general, abundant placental fibrin deposition is a hallmark of pregnancies complicated by intrauterine growth restriction and has been linked to physiological states known to also occur in PM such as ischemia and complement activation [7], [8].

To date, assessment of indicators of coagulation other than fibrin deposition in malaria-infected placentae has been limited. Imamura et al. [9] showed that excessive fibrin deposition in the infected placenta occurs in association with dramatic upregulation of tissue factor (TF), the initiator of the extrinsic coagulation cascade, on infiltrating monocytes. However, the complex dynamics of inflammation, coagulation, and fibrinolysis in the infected placenta, and how these phenomena converge to compromise pregnancy, have not been investigated. To provide evidence that PM induces dysregulated hemostasis, markers of coagulation and fibrinolysis were assessed in placental plasma and tissue derived from women exposed to holoendemic malaria. Furthermore, to identify a potential therapeutic benefit of blocking fibrin formation during pregnancy, *P. chabaudi* AS-infected pregnant mice, which share important immunopathogenic features with human PM [10]–[12] were treated with low molecular weight heparin. The results suggest that dysregulated hemostasis is an important feature of PM and anticoagulant treatment may represent a novel therapeutic avenue for averting poor birth outcomes associated with malaria during pregnancy.

## Materials and Methods

### Ethics statement

All study procedures and instruments involving human subjects, data and sample collection, processing, and testing were approved by the University of Georgia and Centers for Disease Control and Prevention Institutional Review Boards and the Kenya Medical Research Institute Ethical Review Board. All participants provided informed, written consent under the auspices of these approved protocols.

Mouse experiments were performed in accordance with the guidelines and with the approval of the University of Georgia Institutional Animal Care and Use Committee (AUP number A2009 4-070).

### Patient recruitment and sample collection and processing

Parturient women exposed to holoendemic malaria transmission in western Kenya were recruited into a cross-sectional study designed to assess gravidity-dependent, T cell-mediated immune responses to malaria. Recruitment was conducted at New Nyanza Provincial General Hospital, a public referral hospital, in Kisumu from November, 2002 to May, 2004, and subsequently at Siaya District Hospital, a public secondary health facility in Siaya until September, 2008, as described previously [13]. Women of all gravidities with uncomplicated pregnancies and deliveries were recruited randomly from patients admitted to the Delivery Wards of these hospitals. Only women with no health issues other than malaria or human immunodeficiency virus infections were eligible for full participation in the study. In these areas, *P. falciparum* is the predominant infective species. Maternal demographic and clinical information was collected using a standard set of study forms. Infant gestational age was estimated using the modified Dubowitz score, and birth weight in grams was measured within eight hours after delivery. Maternal placental (intervillous) blood (IVB) was collected by the prick method within five minutes of placental expulsion and by perfusion within eight hours of expulsion [14]. In some cases, prick blood was submitted for a complete blood count (COULTER® A^c^.T™ 5diff CP, Beckman Coulter, Miami, FL). EDTA and heparin-anticoagulated prick blood was centrifuged to yield platelet-free plasma and blood cell pellets, both of which were preserved in liquid nitrogen. PM was evaluated by thick and thin IVB smear as described [13]. In addition, the percentage of leukocytes that contained phagocytosed parasite hemozoin (Hz) was enumerated among at least 300 total leukocytes on thick smears. Active PM (PM+) was defined as detection of *Plasmodium falciparum* on thick IVB smears by light microscopy. PM− cases were identified as having no evidence of parasites by IVB thick smear unless otherwise indicated.

All recruited PM+ primigravidae for whom a placental plasma sample and histology section were available were included in the present study. An additional eight cases which lacked placental tissue sections were also included (four with LBW and four with normal birth weight infants). Age and season-matched PM− primigravidae (one to two per PM+ case depending on availability of samples) were selected as controls. Among PM− cases, any women bearing LBW infants were also further age and season-matched with PM− women with normal birth weight infants.

### Human placental histology

Tissue blocks (3 mm×0.5–1 cm) representing the full thickness of the placental disk were collected paracentrally from three areas not subjected to perfusion and fixed with Streck Tissue Fixative (Streck Inc., Omaha, NE). Fixed tissue was paraffin-embedded and 5 µm sections stained with hematoxylin and eosin. All sections were reviewed by one author (JMM) with additional independent evaluation of a subset by a second author (CA). One block was evaluated for 54 samples, two for 108, and three for 10, depending on availability and tissue quality. All sections were approached in a blinded manner and initially assessed at low power (100× magnification) for semi-quantitative scoring of placental fibrin on a scale from 0 to 5. For the extent of fibrin deposition at the basal and chorionic plates, the following scale was used to apply a score to each: none (0), scant (1), minimal extension into intervillous space, at chorionic plate with involvement of stem villi (2), moderate (one observation of fibrin extension from basal plate to involve a terminal villus/section; at least one episode of one stem and surrounding villi involved in the same sub-chorionic fibrin deposit/section; 3), heavy (>1 observation of fibrin extension from basal plate to involve terminal villi/section; several episodes of multiple stem and intermediate villi engulfed in fibrin, consuming 1/3 of low power field at chorionic plate/section; 4), or extensive (>1 observation of fibrin extension from basal plate to involve multiple terminal villi/section; several episodes of multiple stem and intermediate villi engulfed in fibrin, consuming >1/2 of low power field at chorionic plate/section; 5). In the remainder of each section, intervillous and perivillous fibrin was also scored: none (0), scant (small regions of free or perivillous fibrin, generally similar in diameter to terminal villi, observed occasionally; 1), minimal (same as scant except observed in multiple fields; 2), moderate (larger regions of free or perivillous fibrin, with entire villi occasionally completely engulfed, observed in multiple sections, with occasional villi converted to fibrin-type fibrinoid; 3), heavy (very large regions of free fibrin several times the diameter of terminal villi, groups of villi completely engulfed, multiple villi converted to fibrin-type fibrinoid, all observed frequently; 4), or extensive (same as heavy, but with at least one of these criteria in every low power section; 5). The final fibrin score was calculated using the formula: (basal score+chorion score+intervillous score×10)/12, with the intervillous score being heavily weighted to account for the relative observed areas of each. Because some level of fibrin deposition is normal, no placentae were scored 0, and only one scored 1. Presence of Hz was similarly scored at 100× final magnification on a scale of 0 (minimal) to 5 (extensive; very large accumulations found throughout). Individual scores for Hz embedded in fibrin or found within leukocytes were applied using essentially the same criteria. Hz in syncytiotrophoblast was similarly scored from 0 to 5; the highest score was applied when multiple small crystals were observed in multiple fields.

In addition, a subset of samples were selected for additional screening by stereological analysis of histological sections modeled after the method used by Crocker et al. [3]. Because this scoring method and the semi-quantitative method yielded correlative data (Figure S1), the latter was used for data analysis in this report.

### Measurement of cytokines and indicators of coagulation and fibrinolysis in IVB plasma

Placental plasma was tested by ELISA for levels of tumor necrosis factor (TNF), interleukin (IL)-10, plasminogen activator inhibitor (PAI)-1, TF, and soluble intercellular adhesion molecule (sICAM)-1 using matched antibody pairs and recombinant standards in DuoSet ELISA Development Systems from R&D Systems (Minneapolis, MN), or in the case of IL-6 as an OptEIA set from Becton, Dickinson and Company (Franklin Lakes, NJ). Lower limits of detection were 3.9 pg/mL, 1.95 pg/mL, 19.5 ng/mL, 3.9 pg/mL, 1 pg/mL, and 1.95 pg/mL, respectively. Samples testing below the limits of detection were assigned half of these amounts. Thrombin-antithrombin (TAT) complexes were measured according to manufacturer specifications using a Matched-Pair Antibody Set and standards from Enzyme Research Laboratories (South Bend, IN). TAT standards were generated from purified antithrombin and thrombin as per manufacturer's specifications. The efficiency of TAT formation, routinely >92%, was confirmed by measuring residual thrombin activity with the colorimetric substrate S-2238 (Chromogenix; Bedford, MA). TF Pathway Inhibitor (TFPI) was detected using mouse monoclonal antibody (clone 374720; 1 µg/mL), biotinylated goat polyclonal antibody (0.1 µg/mL), and recombinant human TFPI (residues 29–282) as standard (R&D Systems), with a limit of detection of 0.244 ng/mL. Soluble CD163 (sCD163) was detected using monoclonal mouse antibody (clone 215927; 1 µg/mL), biotinylated goat polyclonal antibody (25 ng/mL) and recombinant extracellular domain of human CD163 (residues 41–1045) as standard (R&D Systems), with a limit of detection of 1 ng/mL. D-dimers were measured using mouse monoclonal antibody (clone 015-22-1; 0.5 µg/mL) from Santa Cruz Biotechnology (Santa Cruz, CA), HRP-conjugated mouse monoclonal antibody (clone DD4; 0.25 µg/mL) from Abcam (Cambridge, MA) and human D-dimer standard from Lee Biosolutions (St. Louis, MO), with a limit of detection of 2 ng/mL. TFPI, sCD163 and D-dimer assays were optimized to maximize detection and minimize background. SuperBlock® (Thermo Scientific) was used for blocking and sample dilution in the D-dimer assay; TFPI and sCD163 used 1% bovine serum albumin in Tris-buffered saline.

### Flow cytometry

IVB isolated by perfusion was processed, stained, and analyzed by flow cytometry as previously described [15]. Monocyte levels are presented as percent of CD45+ cells that are CD14+. To obtain monocyte counts, the leukocyte count derived from a complete blood count was multiplied by the percent of CD14+/CD45+ cells.

### Molecular malaria diagnosis

To identify sub-microscopic cases of human PM (PM^sub^), DNA was isolated from 100–200 µL of frozen IVB pellets from all samples found to be PM− by light microscopy using the GE Healthcare (Piscataway, NJ) Illustra Blood Genomic Prep Mini Spin Kit according to manufacturer's specifications. Isolated DNA equivalent to 5 µL of cell pellet was introduced into a PCR reaction targeting a recently described multi-copy sequence (220 basepair amplicon – Pfr364) unique to the *P. falciparum* genome using Alt-Forward (5′-CCG GAA ATT CGG GTT TTA GAC) and Alt-Reverse (5′-GCT TTG AAG TGC ATG TGA ATT GTG CAC) primers as described by Demas et al [16]. Genomic DNA similarly isolated from two non-malaria-exposed American volunteers was included as a negative control and DNA from placental blood of a microscopy-confirmed PM case was included as a positive control in all reactions.

### Mice, parasites, anticoagulant therapy, and clinical assessment

C57BL/6J (B6) mice were bred and maintained at the University of Georgia Animal Resources facility as previously described [10], [12]. Experimental breeding, parasite maintenance and infections, and monitoring and sampling of experimental mice were accomplished using a previously established protocol [10]. Briefly, the day on which a vaginal plug was observed in timed mated eight- to twelve-week-old, female C57BL/6 mice was considered day zero of pregnancy, experiment day (ED) 0. Intravenous infections were initiated on this day with 10^3^ infected red blood cells. Mice were observed during ED 6 to 12; parasitemia was monitored by counting 1×10^3^ erythrocytes in four high-power fields on Giemsa-stained tail blood thin smears. Hematocrit was used as a measure of anemia. Blood collected from the tail vein into heparinized capillary tubes was centrifuged in a microhematocrit centrifuge and percent hematocrit was calculated according to the following: (volume of packed erythrocytes)/(total blood volume)×100. Euthanasia was accomplished via CO_2_ asphyxiation followed by cardiac puncture. Blood was collected into Microtainer K_2_EDTA tubes (Becton Dickson, Franklin Lakes, NJ, USA).

Infected pregnant (IP) mice were administered 1000 IU/kg low molecular weight heparin (LMWH; Calbiochem, San Diego, CA, USA) or enoxaparin (Lovenox, Sanofi-Aventis, Bridgewater, NJ, USA) subcutaneously, via the scruff of the neck, twice daily from ED 6 through ED 12. The treatment protocol was confirmed to induce no adverse effects in five uninfected pregnant (UP) mice (data not shown). Initial low dose regimens (70 IU/Kg, 120 IU/Kg, and 220 IU/Kg, given once every 24 hours or once every 12 hours via intraperitoneal or subcutaneous administration to IP mice), guided by studies of spontaneous abortion in mice [17], were abandoned due to lack of efficacy. Because two low molecular weight heparins were used for anticoagulant treatment, LMWH refers to the research grade sample, while enoxaparin refers to the FDA approved drug. Infected non-pregnant (INP) and uninfected pregnant (UP) control mice were given sham subcutaneous injections of PBS.

Active abortion and embryo resorption were scored antemortem and at necropsy as previously described [10]. In active abortion cases, all embryos were scored as non-viable, regardless of the state of the remaining embryos upon necropsy. If upon necropsy, evidence of active expulsion was observed, all embryos were scored non-viable. Only embryos of females that did not demonstrate active abortion were assessed for viability. Embryos exhibiting intra-embryonic or placental hemorrhage were scored as non-viable. Uteri from UP, IP, and IP LMWH-treated mice were harvested on ED 12 and fixed in 4% paraformaldehyde overnight.

### Mouse conceptus histology

Tissues were processed and stained as above for human placentae. Sections were evaluated independently by two authors (JWA and TN), scoring for necrosis and loss of architecture of the placental layers and embryo.

### Cell Culture and parasite stimulation

The outbred Swiss Webster mouse trophoblast cell line, SM9-1, was generously provided by Dr. Joan Hunt (University of Kansas Medical Center, Kansas City, KS) and maintained in RPMI 1640 complete (10% fetal bovine serum, 2 mM L-glutamine, 100 U/ml penicillin and 100 µg/ml streptomycin, 1 mM sodium pyruvate, 1.75 µM 2-mercaptoethanol) as described [18]. *P. chabaudi* AS-infected erythrocytes were recovered from A/J mice and used to stimulate SM9-1 cells as described previously [11]. Briefly, *P. chabaudi* AS-iRBCs were recovered from A/J mice, washed and loaded onto a 74% Percoll (Sigma-Aldrich, St. Louis, MO, USA) density gradient. Following centrifugation at 1500× *g* for 20 min at 4°C, the top interface, which routinely contained >85% parasitized RBCs (mature trophozoite and schizont stages), was harvested and washed. Uninfected RBCs were collected in the same fashion from uninfected A/J mice and loaded onto a 90% Percoll® gradient, centrifuged at 1500× *g* for 20 min at 4°C, and the top interface collected. Three million SM9-1 cells were plated (10^6^ cells/mL RPMI) in Corning Costar® 6 well culture plates (Sigma-Aldrich, St. Louis, MO, USA) and at ∼80% confluence were co-cultured with either iRBCs or uninfected RBCs at a cell to red blood cell ratio of 10∶1 in a 37°C incubator with an atmosphere of 5% CO_2_. Cells were harvested at times 0, 2, 4, 6, and 8 hours post exposure via trypsinization for RNA isolation.

### RNA Isolation, cDNA generation, and quantitative polymerase chain reaction (qRT-PCR)

Uteri from ED 10 IP and UP mice were removed and conceptuses isolated and homogenized in a TissueLyser II (Qiagen, Valencia, CA, USA). Conceptus total RNA from homogenates or SM9-1 cell RNA was reverse transcribed, subjected to qRT-PCR, and analyzed as described [12]. Briefly, RNA was reverse transcribed using High-Capacity cDNA Reverse Transcription and DNA-free Kits (Applied Biosystems, Carlsbad, CA, USA) or RNeasy® Plus Mini Kit (Qiagen, Valencia, CA, USA) as described by the manufacturers' protocols. Real-time PCR was performed on and analyzed with an ABI 7500 Real-Time PCR System (Applied Biosystems, Carlsbad, CA, USA) using Maxima® SYBR Green with passive reference (Fermentas, Glen Burnie, MD, USA). Target gene expression levels were normalized to the internal 18S signal and represented as relative expression calculated by the ΔΔCT method. Specific PCR primer pairs (Table 1) were used for the following mouse genes of interest: coagulation factor III (*F3*); tissue factor pathway inhibitor (*Tfpi*); thrombomodulin (*Thbd*); coagulation factor II (thrombin) receptor (*F2r*); coagulation factor II (thrombin) receptor-like 1 (*F2rl1*); protein C receptor, endothelial (*Procr*); serine peptidase inhibitor member 1 (*Serpine1*); 18S ribosomal RNA (18S). Primers were created using Primer Express (Applied Biosystems, Carlsbad, CA, USA) and oligonucleotides were generated by (Eurofins MWG Operon, Huntsville, AL, USA).

**Table 1:** Primers used in quantitative real time PCR expression analysis.

Target	Accession Number	Primer Sequences	
*F3* (TF)	NC_000069.5	FWD	5′-CCA CCA TCT TTA TCA TCC TCC T-3′
		REV	5′-AGC CTT TCC TCT ATG CCA AGC-3′
*Tfpi* (TFPI)	NC_000068.6	FWD	5′-CCA GAG AAC CAC AGC ACC AC-3′
		REV	5′-CAA GGG CAA GAG GCA GAT-3′
*Thbd* (TM)	NC_000068.6	FWD	5′-TAG GGA AGA CAC CAA GGA AGA G-3′
		REV	5′-GAG AGA GAGA GGA GAG GAG AGG-3′
*F2r* (PAR-1)	NC_000079.5	FWD	5′-TAC ATA ACA CCC CTT CGG CTA T-3′
		REV	5′-AAC ACA CCT TTCTCC TCT CGT C-3′
*F2rl1* (PAR-2)	NC_000079.5	FWD	5′-CAC CTG GCA AGA AGC CTA AG-3′
		REV	5′-CCC AGG GTT ACT GAC GCT AA-3′
*Procr* (EPCR)	NC_000068.6	FWD	5′-CAT CGG AGT TAC AAA GGG CG-3′
		REV	5′-CCC AGA ACT CCA GGA TGT TGA-3′
*Serpine1* (PAI-1)	NC_000071.5	FWD	5′-GGC ACA ACA CTT TCA TTC AGC-3′
		REV	5′-CGA CTT TTC TTA CAC CCT TTC C-3′
18s rRNA	NR_003278	FWD	5′-CCA TCC AAT CGG TAG TAG CG-3′
		REV	5′-GTA ACC CGT TGA ACC CCA TT-3′

### Western blotting

Pooled conceptuses (at least four) from two C57BL/6J mice at ED 10 and 11 and their appropriate uninfected controls (two at each time point) were homogenized and proteins isolated, processed and detected by western blot as described [19]. Membranes were probed with mouse monoclonal antibody for the fibrin beta chain (ADI 350; American Diagnostica, Stamford, CT) and mouse monoclonal antibody for beta-actin (clone AC-15; Sigma-Aldrich, St. Louis, MO) as a loading control. Proteins were detected with affinity purified horse anti-mouse horseradish peroxidase (HRP) conjugate from Cell Signaling (Beverly, MA).

### Statistical analysis

Data analyses were performed using GraphPad Prism 5 Software (La Jolla, CA, USA) and SAS version 9.2 Software (Cary, NC, USA). Correlation analysis was done using Spearman's test and 2×2 contingency tables were used for testing differences between proportions. The significance of difference of group means in the case of normally distributed data were compared via t tests for pairwaise comparisons or one-way ANOVA with Tukey's Post-hoc Multiple Comparison Test for multiple group comparisons. Non-normally distributed data were analyzed by non-parametric, Mann Whitney test for pairwise comparisons and Kruskal-Wallis test with Dunn's Multiple Comparison post-test for multiple group comparisons. Multiple linear regression analysis was performed including interaction terms for dependent variables and selected parameters. Statistical significance was not observed for select parameters, notably low birth weight and interactions involving low birth weight. Non-parametric human data are plotted using log_10_ scales for ease of viewing but were not log-transformed. Proportional analysis was accomplished via two-sided Fisher's exact test. Values of *P*≤0.05 were considered to be significant.

## Results

### Study participant characteristics

The investigation was restricted to primigravidae since they are known to have the highest risk for PM, malaria-associated LBW and prematurity, and PM-associated placental pathology, including fibrin deposition [5], [6]. Based on light microscopic evaluation of intervillous blood thick and thin smears, samples from a total of 79 PM+ and 114 PM− women were available for inclusion in the study. Microscopic diagnosis of PM by blood smear has been shown to have low accuracy; in a previous analysis of a subset of samples from this study population, approximately half of all PM cases were detectable only by PCR [13]. Targeting a newly described high-copy target in the *P. falciparum* genome using PCR [16] revealed that 30 of 108 (28%) smear-negative samples were submicroscopically infected (PM^sub^). Table 2 lists relevant clinical and sociological attributes of all participants; for the purposes of these summary characteristics, smear-negative participants for whom PCR testing was not possible (n = 6) were included with the PM− group. Relative to PM− women, a greater proportion of PM+ women bore LBW infants, and mean birthweights among the latter were significantly lower than among women with no active infection; to some extent this was expected based on the sample selection scheme. Most cases of LBW were due to fetal growth restriction since ≤5% of infants in each group were born earlier than 36 weeks gestation. PM+ women had lower hemoglobin levels than the other groups and significant levels of hemozoin (Hz)-bearing phagocytes on IVB thick smears. Although approximately half of all women reported use of sulfadoxine/pyrimethamine during pregnancy, more than 90% of all women had evidence of current or past PM (malarial hemozoin in fibrin observed by histology).

**Table 2:** Clinical and sociological attributes of human participants.

Group^a^	PM− (84)	PM+ (79)	PM^sub^ (30)	*P*
% Luo ethnicity	84* (76)	97* (76)	89 (28)	0.005*
Age (years)	19±3 (75)	19±3 (77)	19±3 (28)	NS
Maternal hemoglobin	11.7±2.2 (49)	11.0±1.9† (47)	12.5±2.4† (22)	0.018
Gestational age (weeks)	38±2 (80)	38±1 (75)	38±1 (30)	NS
% preterm delivery^b^	5.0 (80)	1.3 (75)	3.3 (30)	NS
Birthweight (g)	3120±458† (80)	2922±428† (79)	3093±379 (30)	0.013
% LBW^c^ infant	13.8* (80)	26.6* (79)	13.3 (30)	0.033*
Apgar 10	10±0 (75)	10±0 (76)	10±0 (28)	NS
% male infant	58 (76)	53 (77)	50 (28)	NS
% SPuse^d^	54.5 (77)	49.4 (77)	50.0 (28)	NS
placental % parasitemia^e^	-	0.72 (76)	-	-
% Hz-bearing WBCs^f^	0.0±0.1‡	7.8±10.9‡,§	0.0±0.0§	<0.0001
% with Hz in fibrin^g^	82 (79)*	99 (71)*	96 (28)	0.0007*
% with Hz in WBCs^h^	44 (79)*^,#^	90 (71)*	61 (28)^#^	≤0.0013*^,#^

Data are presented as mean ± standard deviation or percentage with sample size in parentheses.

a PM = placental malaria; PM^sub^ indicates microscopy negative, PCR positive participants.

b defined as <36 weeks gestation.

c LBW = low birth weight.

d SP = reported use of sulfadoxine-pyrimethamine.

e geometric placental parasitemia from intervillous blood thin smear.

f Hz = hemozoin; WBC = white blood cell; indicates percent of WBCs bearing hemozoin on intervillous blood thick smear.

g indicates chronic or past infection as evidenced by the presence of any Hz in fibrin observed by histology.

h indicates chronic or past infection as evidenced by the presence of any Hz in intervillous WBCs observed by histology.

Statistics by one-way ANOVA (*P* values shown in table) with Tukey's post-hoc test for continuous variables:

† *P*<0.05,

‡,§ *P*<0.001.

Fisher's exact test was used for pairwise comparison of proportions (*P* values shown in table with *^,#^ symbols indicating significant comparisons).

### Blood smear-positive PM is associated with inflammatory responses and dysregulated hemostasis in IVB

**Figure 1:**
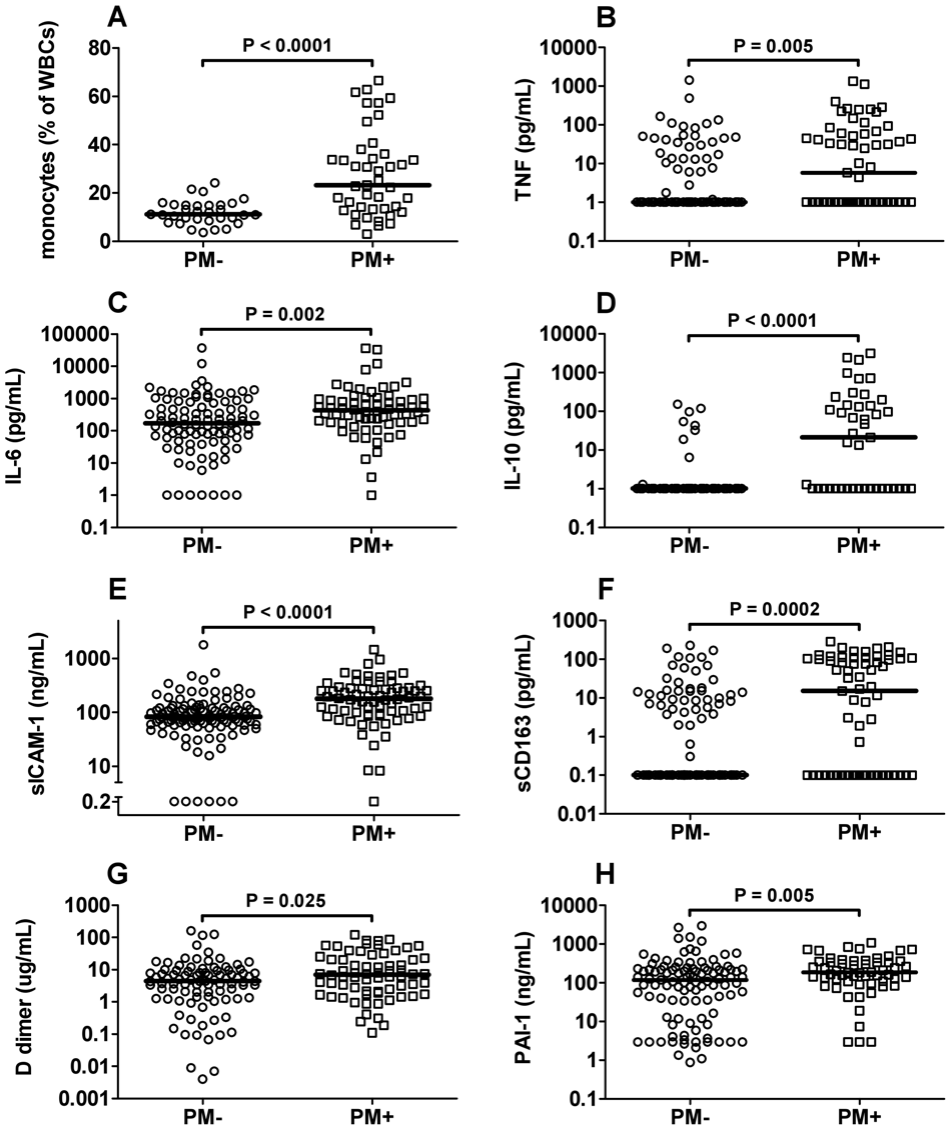
PM is associated with inflammatory responses, increased markers of coagulation, and suppressed fibrinolysis. (A) Monocyte levels detected in IVB by flow cytometry. (B–H) TNF, IL-6, IL-10, sICAM-1, sCD163, D-dimers and PAI-1 detected in IVB by ELISA. Samples in all panels were stratified by presence or absence of microscopically evident placental parasitemia. Bars represent the median.

In initial analyses, participants were grouped according to results of IVB blood smears. Thus, PM^sub^ women were combined with PM− women. As evidenced by flow cytometric evaluation, PM+ women had significantly higher levels of IVB monocytes than PM− women (Figure 1A). Among those for whom a complete blood count of IVB was available, PM+ women had significantly higher monocyte counts (median (quartiles): 4155 (2206, 7888)×10^3^/µL; n = 17) than PM− women (1849 (953, 2640)×10^3^/µL; n = 10; *P* = 0.011). Several soluble markers of inflammation, TNF, IL-10, IL-6, sCD163, and sICAM-1, were also significantly elevated in PM+ placental plasma (Figure 1B–F).

Semiquantitative assessment of histological sections for placental fibrin revealed that PM+ women had more deposition than their PM− counterparts (mean ± SD: 3.4±0.9 vs 3.7±0.9; *P* = 0.019), although the difference was subtle. Evaluation of IVB plasma for markers of active coagulation (degradative products of fibrin, D-dimers) and suppression of fibrinolysis (PAI-1) by ELISA revealed increases for both in association with microscopically evident PM (Figure 1G, H). Soluble TF, TFPI and TAT complex levels did not differ between these two groups (data not shown).

### Submicroscopic PM induces dysregulated hemostasis in IVB

**Figure 2:**
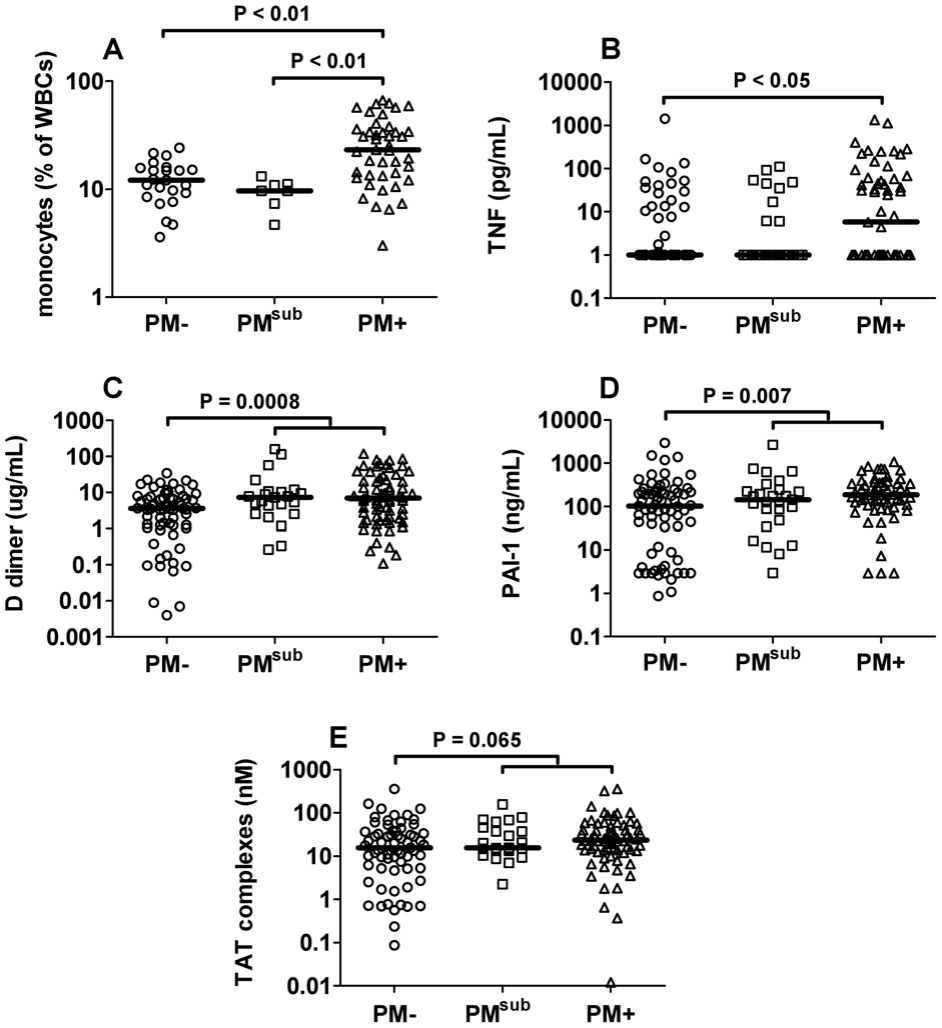
Submicroscopic PM does not induce inflammatory immune responses, but does dysregulate hemostasis. (A) Monocyte levels in IVB as detected by flow cytometry. (B) TNF levels in IVB. (C) D-dimer levels in IVB. (D) PAI-1 levels in IVB. (E) TAT complex levels in IVB. TNF and coagulation/fibrinolysis markers were measured by ELISA. Statistical results in panels C, D and E represent analysis of submicroscopic (PM^sub^) and microscopic (PM+) groups combined versus PM− samples. Bars represent the median.

To determine the extent to which submicroscopic PM may influence hemostatic function in the placenta, participants were next stratified based on the results of both IVB blood smear and PCR evaluation for PM. Although PM^sub^ samples did not show placental inflammatory infiltrate (Figure 2A) and only PM+ samples had elevated TNF levels relative to PM− placentae (Figure 2B), D-dimer and PAI-1 levels in PM^sub^ placentae clearly grouped with the PM+ samples (Figure 2C, D). Furthermore, similar analysis of combined PM^sub^ and PM+ samples revealed a tendency for enhanced TAT complex production in association with the presence of placental *P. falciparum* relative to uninfected samples (Figure 2E). Despite these observations, fibrin deposition within PM^sub^ placentae was comparable to that in (PCR-confirmed) PM− women (mean ± SD: 3.3±0.9 vs 3.4±0.9; *P*>0.05), suggesting some other factor may be required for enhanced placental fibrin deposition with microscopically evident PM.

### Placental hemostatic dysregulation correlates with PM intensity, inflammation and fibrin deposition

**Figure 3:**
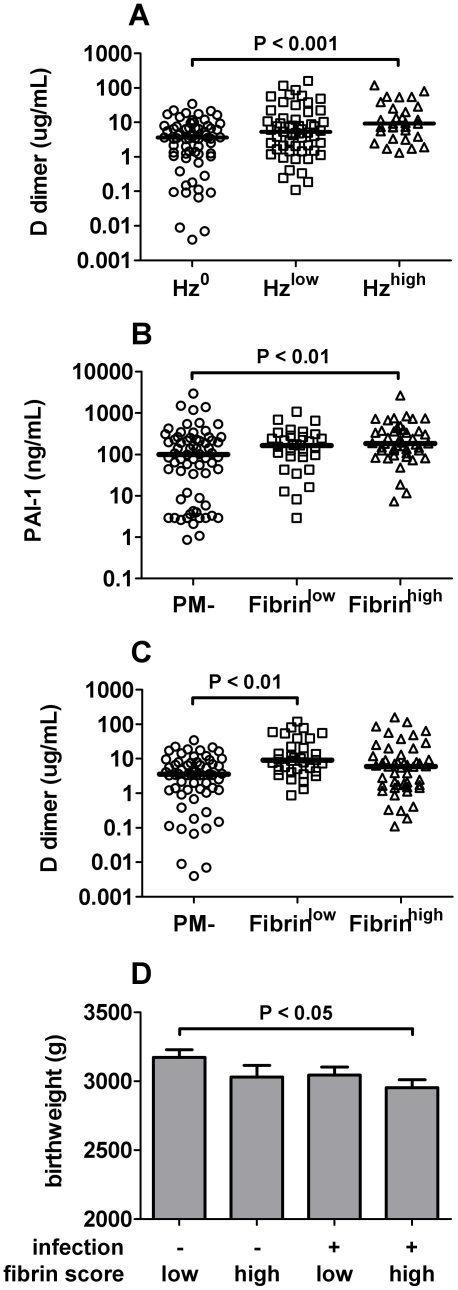
Chronic PM and high placental fibrin deposition are associated with dysregulated hemostasis and reduced birth weight. (A) D-dimer levels measured by ELISA in IVB in PCR-confirmed PM− placentae with no leukocytes bearing Hz on a Giemsa-stained IVB thick smear (Hz^0^) and in PM^sub^/PM+ women with Hz in <5% (Hz^low^) or ≥5% of IVB leukocytes (Hz^high^). PM+ samples with no Hz in leukocytes (n = 6) were excluded from the analysis. (B, C) PAI-1 and D-dimer levels measured in IVB by ELISA in PCR-confirmed PM− placentae and in PM^sub^/PM+ placentae with fibrin score≤3.4 (Fibrin^low^) or >3.4 (Fibrin^high^), cut-offs defined by the mean fibrin score in PM− cases. (D) Birthweights (mean ± SEM) stratified by fibrin score and infection status in PCR-confirmed PM− and PM^sub^/PM+ cases. Bars represent the median in panels A–C.

To evaluate parameters other than parasitemia associated with dysregulated hemostasis, soluble coagulation and fibrinolysis parameters were assessed as a function of inflammatory markers, placental histological features, and a marker for PM intensity, the percent of phagocytic cells on an IVB thick smear that contain Hz [20]–[22]. D-dimer levels in IVB from PM+/PM^sub^ samples were significantly higher in the presence of elevated levels of Hz-bearing phagocytes (>5% of all leukocytes) relative to PM− samples with no Hz (Figure 3A). In addition, within the population as a whole, levels of PAI-1, which suppresses fibrin degradation, were weakly positively correlated with the presence of Hz-bearing WBCs observed by histology (r = 0.213, *P* = 0.009). Likewise, histological fibrin score was positively correlated with percent monocyte levels in IVB (r = 0.260, *P* = 0.026). A number of other positive correlations observed among the coagulation and fibrinolysis parameters and with inflammatory factors are summarized in Table 3. Finally, stratification of PM+/PM^sub^ cases into two groups defined by fibrin score revealed that PAI-1 was significantly elevated in those with high fibrin accumulation (score>3.4, the observed mean among PM− cases) relative to PM− samples (Figure 3B), but not in those with low fibrin accumulation (score≤3.4). In contrast, D-dimers were higher in PM/PM^sub^ cases with low fibrin accumulation compared to PM− placentae, a relationship not observed in the context of high fibrin deposition (Figure 3C).

**Table 3:** Correlations among hemostatic parameters and inflammatory markers.

Variables	D-dimer	TAT complex	PAI-1	TFPI	TF
D-dimer	-	-	-	-	NS
TAT complex	NS	-	-	-	NS
PAI-1	0.258**	0.518***	-	-	NS
TFPI	NS	0.180*	0.259**	-	0.321***
TNF	NS	NS	NS	0.190*	NS
IL-10	0.255**	NS	NS	NS	NS
IL-6	0.287***	0.370***	0.529***	0.211**	0.226*
sCD163	0.235**	0.228**	0.421***	NS	NS

Data represent Spearman r and summary of *P* values with * *P*<0.05, ** *P*<0.01, *** *P*<0.001, and NS = not significant.

### A role for dysregulated hemostasis and placental fibrin deposition in pregnancy outcome

Previous studies have shown a connection between fibrin deposition in the infected placenta and LBW, in one case, in association with premature birth [3], [6]. Although due to study design poor birth outcomes were underrepresented in this study, it was of interest nonetheless to examine the impact of dysregulated hemostasis and fibrin deposition on birth outcomes. High fibrin deposition score was not associated with an alteration in weeks of gestation at delivery, and none of the hemostatic parameters (TAT complexes, D-dimers, PAI-1, TFPI, fibrin score) was correlated with gestational age (data not shown). Multivariate analysis of the impact of D-dimers, TAT complexes, PAI-1 and TFPI on birthweight (both as a categorical variable, LBW versus normal birth weight, and a continuous variable) while controlling for PM infection also did not reveal any statistically significant associations. However, comparison of birth weights as a function of PM and fibrin score showed that infants born to PM+/PM^sub^ cases with a high fibrin score had significantly lower birth weights than those born to PM− women with a low fibrin score (Figure 3D). Indeed, across the four groups (PM− low and high fibrin, and PM+/PM^sub^ low and high fibrin) a trend toward decreasing birth weight was evident (test for linear trend, *P* = 0.021).

### Plasmodium chabaudi infection supports the upregulation of coagulation-associated genes

**Figure 4:**
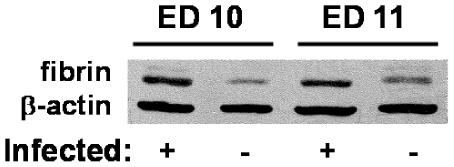
Fibrin deposition is enhanced in conceptuses from IP mice. Total protein from pooled IP and UP conceptuses probed with fibrin antibodies on western blot with control β-actin antibody as a loading control (as described in methods).

**Figure 5:**
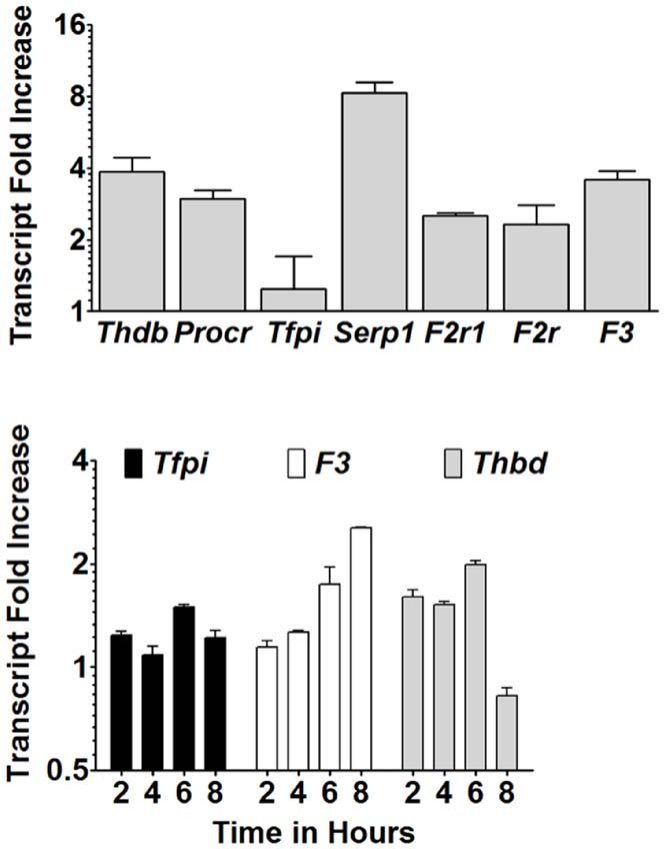
Coagulation factor gene expression is elevated in IP mice and malaria-exposed murine trophoblasts. (A) RNA was isolated from conceptuses removed from ED 10 UP (n = 5) and IP (n = 6) mice. Primers specific for the genes indicated were utilized to measure cDNA expression levels in IP relative to UP mice. Data are normalized against murine *18S* RNA. Data are expressed as the ratio of fold increase in IP mice to that of UP mice ± SEM. (B) SM9-1 trophoblasts were stimulated with *P. chabaudi* AS-iRBCs and RNA isolated over the time course indicated. QRT-PCR was conducted as in panel A. Data are expressed as the ratio of fold increase relative to time matched SM9-1 trophoblasts stimulated with uninfected RBC ± SEM and are representative of four separate experiments.

B6 mice infected with *P. chabaudi* AS in early pregnancy fail to maintain viable embryos, with conceptus failure beginning at ED 10 and complete pregnancy loss by ED 12 [10]. Inflammatory mediators of pathogenesis in this model have been well described [10]–[12]; however, the contribution of coagulation to the associated pathology has yet to be comprehensively examined. Expression of TF, the initiator of the extrinsic coagulation cascade, is elevated on trophoblasts in these mice [11] and fibrin deposition is elevated in IP conceptuses (Figure 4). To assess the extent to which *P. chabaudi* AS infection impacts the expression of coagulation factors during pregnacy, conceptuses were recovered from IP mice at ED 10, when embryo loss commences in this model [10], [11], and expression of several genes whose products are involved in hemostasis measured (Figure 5). The genes *Thbd*, *F3*, and *Serpine1* (encoding thrombomodulin, TF, and PAI-1, respectively) were upregulated 3.9-, 3.6-, and 8.3-fold, respectively. Both *F2r* and *F2rl1* (protease-activated receptors (PAR) 1 and 2) expression levels were double those in UP mice (2.3 and 2.5 fold, respectively), and *Procr* (protein C receptor) exhibited a 3-fold increase. *Tfpi* message was unchanged (1.2-fold) with wide relative variability.

To investigate the extent to which trophoblasts might be responsible for these changes in coagulation factor expression, SM9-1 trophoblasts were stimulated *in vitro* with *P. chabaudi* AS-iRBCs and transcripts for *F3*, *Tfpi*, and *Thbd* assessed by qRT-PCR. Similar to the evidence from IP mice, malaria-exposed SM9-1 trophoblast displayed the ability to support coagulation by marked increases of *F3* transcription (Figure 5B), which steadily increased, peaking at 8 hours post-iRBC exposure with a mean fold increase of 2.5±0.1. *Thbd* transcripts were increased at early time points, but in contrast to the observation in ED 10 conceptuses, were suppressed at 8 hours. Interestingly, a similar observation was made in human primary trophoblasts exposed to chondroitin sulfate A-adherent *P. falciparum* iRBCs (data not shown). Consistent with the *in vivo* pattern, SM9-1 *Tfpi* transcript levels were insensitive to iRBC exposure.

### LMWH therapy improves midgestational embryonic survival

**Figure 6:**
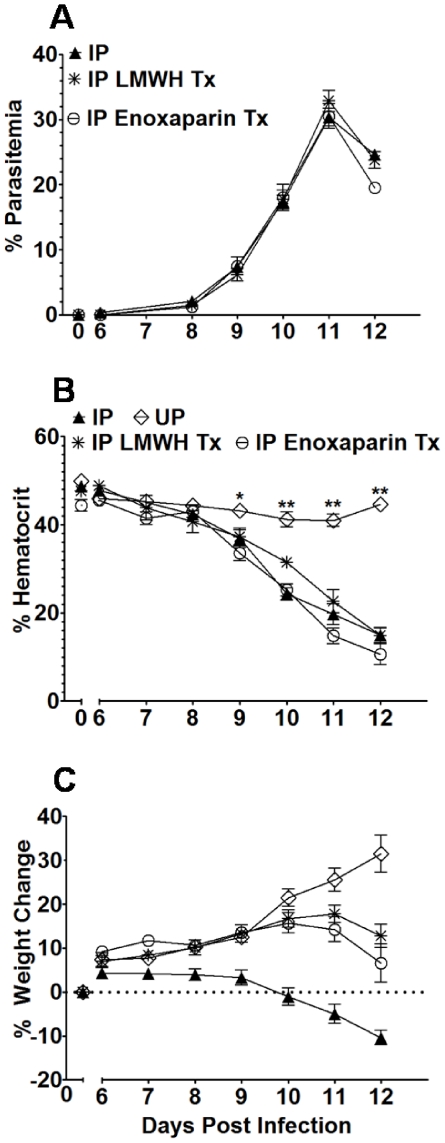
LMWH and enoxaparin therapy improve midgestational body weight. (A–C) Percent parasitemia, hematocrit and change in body weight of UP (n = 19), IP (n = 14), IP LMWH-treated (n = 11), and IP enoxaparin-treated (n = 5) mice are shown. Clinical metrics were measured on ED 0 and from ED 6 to 12. Data represent mean ± SEM. *P<0.0033; **P<0.0001.

**Figure 7:**
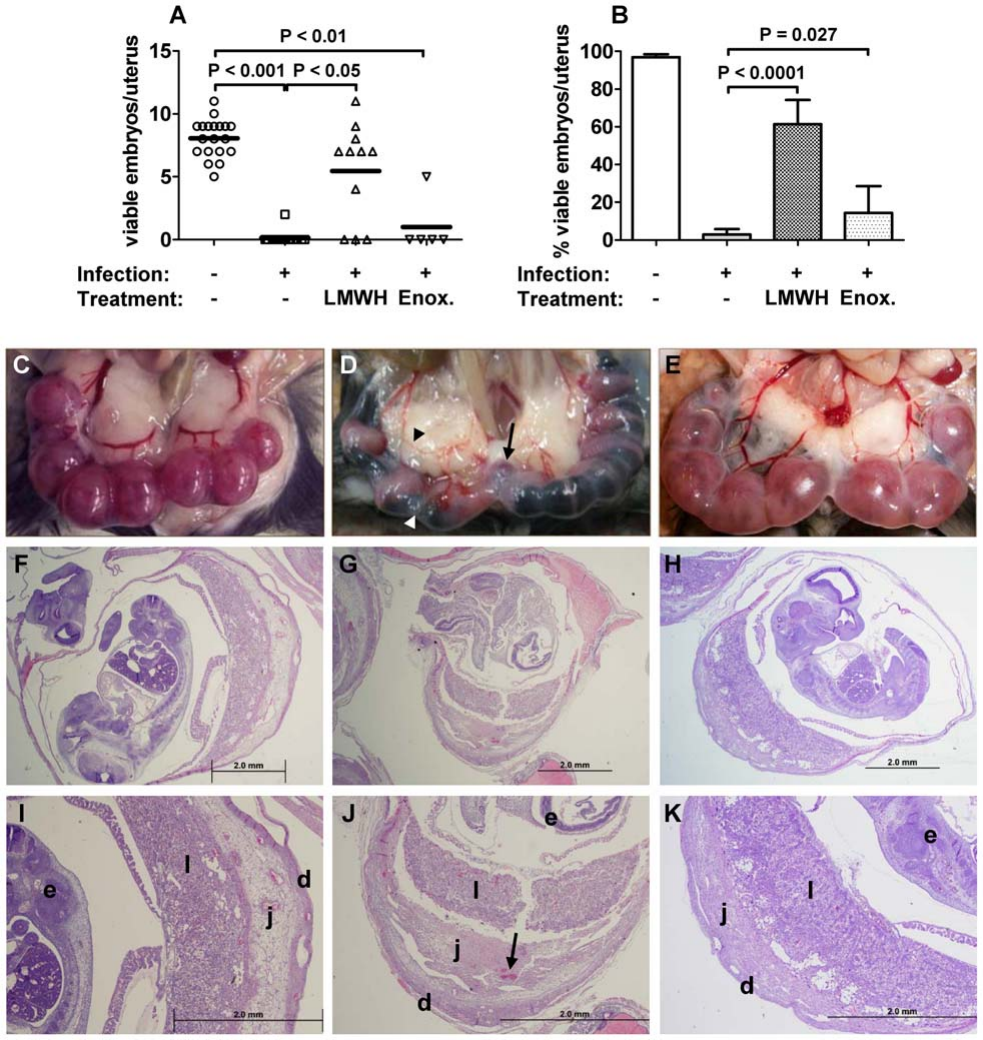
LMWH treatment improves midgestational embryo survival in IP mice. (A) Viable embryos per mouse among UP (n = 18), IP (n = 15), IP LMWH-treated (n = 11), and IP enoxaparin-treated mice (n = 5) on ED 12. Bars represent the mean. (B) Mean (± SEM) viable embryos as a proportion of total embryos within each group as described in panel A. *P<0.0001, **P = 0.0008, ***P = 0.0270. (C) Gross pathological view of UP uterus. (D) Gross pathological view of IP uterus, showing active embryonic expulsion (arrow), diminished vascularization (black blunt arrow), and intrauterine hemorrhage (white blunt arrow). (E) Gross pathological view of LMWH-treated IP uterus with one resorption (arrow). (F, I) Hematoxylin and eosin (H&E)-stained thin section of a UP conceptus. (G, J) H&E-stained thin section of an IP conceptus; arrow indicates fibrin deposition. (H, K) H&E-stained thin section of an IP LMWH-treated conceptus. Enlargements (panels I, J and K) delineate the three principle regions of the murine placenta, decidua (d), junctional zone (j), labyrinth (l), and also identify the embryo (e). Gross macroscopic pictures were taken with a Kodak Easyshare DX7630 digital camera at 6 MP. Micrographs were captured on an Olympus BX41TF light microscope using an Olympus D70 digital camera. Panels F, G, and H depict magnification with a 2× objective and panels I, J, and K with a 4× objective. Images were resized, cropped as appropriate, and in some cases brightened using GNU Image Manipulation Program v2.6.

To assess the role coagulation plays in pregnancy loss in this model, various anticoagulants were administerred to IP mice. The feasibility of such an approach is supported by the proven efficacy of anticoagulant treatment in improving outcomes in patients suffering from sepsis, and the safety and benefit of anticoagulant therapy in women experiencing recurrent abortion [17], [23]–[27]. Mice treated with research grade LMWH and enoxaparin exhibited the same infection kinetics as untreated IP mice (Figure 6A). Hematocrit values for the IP-treated and -untreated groups remained similar throughout the monitoring period, differing significantly from UP mice beginning on ED 10 (Figure 6B). In contrast, the IP-treated groups paralleled the weight gain observed in UP mice up to ED 9, whereas untreated IP mice failed to gain appreciable weight and were the only group to fall below their starting weight (Figure 6C). This weight loss directly correlated with their inability to maintain viable pregnancies at ED 12 (Figure 7A, B). LMWH-treated IP mice demonstrated the most therapeutic benefit, continuing to gain weight until ED 11, with a slight (5%) loss from ED 11 to 12 (Figure 6C). Correspondingly, embryo survival in these mice at ED 12 was not different from UP mice (Figure 7A, B), although 3/11 LMWH-treated mice had evidence of active abortion whereas no UP mice (0/18) aborted. Enoxaparin-treated mice exhibited an intermediate phenotype, continuing to gain weight up to ED 10 (Figure 6C), but having poor embryo viability similar to untreated IP mice (Figure 7A, B); in this group 4/5 mice displayed active abortion at ED 12. Overall, 61% of LMWH-treated and 14% of enoxaparin-treated embryos survived compared to 3% in the untreated IP group (versus LMWH, *P*<0.0001; versus enoxaparin, *P* = 0.0270). However, neither LMWH nor enoxaparin treatment afforded viability to the level observed in UP mice (97%; Figure 7B).

Differences in midgestational embryo survival among the treatment groups were clear in gross pathological and histological examinations (Figure 7C–K). Pregnancy loss associated with active abortion was in some cases evident antemortem, most common among untreated IP mice. Active abortion was also evident at necropsy, with embryos engaged at or passing through the open cervix (Figure 7D, arrow); in such cases, all embryos were considered to be non-viable, with most undergoing resorption and/or exhibiting significant intra-embryonic and intrauterine hemorrhage (Figure 7D, asterisk). Embryos from UP mice at ED 12 showed no hemorrhage, no active expulsion and only five resorptions out of 146 embryos examined (Figure 7B, C). Histological examination of UP mice demonstrated that the three principal layers of the placenta, the decidua, junctional zone, and labyrinth, exhibited normal and intact architecture, with no evidence of embryonic necrosis (Figure 7F, I). In contrast, untreated IP mice demonstrated significant loss of placental architecture (Figure 7G, J), with considerable necrosis and large deposits of fibrin within the junctional zone (Figure 7G, arrow). These features were absent in IP LMWH-treated mice (Figure 7E), which instead showed gross features similar to UP mice (Figure 7C). Relative to untreated IP mice, few intra-embryonic hemorrhages were observed and resorption events were significantly reduced in LMWH-treated mice (Figure 7B, E). Moreover, histological examination revealed a preservation of placental architecture and embryonic stability in these treated animals (Figure 7H, K). Enoxaparin-treated IP mice did not experience a significant increase in viability due to the high number of active expulsions occurring by ED 12 (Figure 7A, B). Gross pathological and histological examinations revealed a similar pattern of intrauterine hemorrhage and resorptions as that of untreated IP mice, with the exception of a single mouse not undergoing active expulsion; this mouse displayed a reduced number of resorptions (2/7 total embryos resorbing) and intact placental architecture in the viable conceptuses (data not shown).

## Discussion

Although there is ample evidence that fibrin deposition is a remarkable and consistent pathological feature of the malaria–infected placenta [5] and is associated with poor birth outcomes [3], [6], only one study [9] has been devoted to identifying the molecular mechanisms that underlie this pathology. Likewise, several studies have revealed a clear role for dysregulated hemostasis in the pathogenesis of severe malaria in non-pregnant patients [28], yet this phenomenon has not been examined in malaria-exposed pregnant women. In this investigation, we brought these two concepts together to demonstrate that PM induces dysregulated hemostasis, and thus provide an expanded functional explanation for the excessive fibrin accumulation found in the infected placenta.

The present study shows that both active coagulation and suppressed fibrinolysis are evident at the placental level in association with submicroscopic and blood smear-evident PM in primigravid women. Interestingly, enhanced D-dimer and PAI-1 levels but not fibrin deposition were observed in PM^sub^ cases. Likewise, these placentae had little PM-associated inflammation, suggesting that while low density, mild infection can promote procoagulant and antifibrinolytic responses, only in cases of chronic infection does coagulopathy manifest. Indeed, the well described inflammatory response to PM, which is most notable and common in first pregnancies [4], was evident only in microscopy-positive PM cases together with increased fibrin deposition. Moreover, D-dimer levels were higher and PAI-1 levels positively associated with increased levels of Hz-bearing leukocytes, an important feature of pathogenic PM [29]. High levels of PAI-1 in those infected cases with the highest levels of fibrin deposition suggest that suppressed fibrinolysis and not solely activation of coagulation underlies PM-associated coagulopathy. Together, these observations are consistent with extensive literature that describes the “inflammation-coagulation cycle” in other disease states, in particular, bacterial sepsis [30], and, with relevance for pregnancy, preeclampsia [31].

Proinflammatory responses induced in bacterial sepsis lead to dysregulated hemostasis and hypercoagulation, with a central role for TNF-induced expression of TF [32]. IL-6 is also critical in stimulating TF expression and activation of coagulation [33], [34]. As confirmed here, TNF is significantly upregulated in the malaria-infected placenta [35], and we show for the first time a significant increase in IL-6 expression with PM. TNF directly induces TF expression on the syncytiotrophoblast *in vitro* [36], and upregulated TF expression on this cell, and more so on monocytes, is evident in malarious placentae [9]. In turn, TF expression on monocytes and endothelial cells is associated with increased production of proinflammatory cytokines, including IL-6 and TNF [37]. It is interesting that of the panel of inflammatory markers measured here, only IL-6 expression levels positively correlated with all of the soluble coagulation parameters; determining the source and initiating stimuli of placental IL-6 will therefore be of considerable interest for future studies. PARs expressed by trophoblast [38] and inflammatory cells in the IVB, when cleaved by coagulation proteases, could contribute to this secretion of proinflammatory cytokines [39], [40]. Thus, as in sepsis, PM-induced inflammatory responses, potentially driven in part by trophoblast [12], [19], [41], may activate coagulation in the placenta, thus perpetuating a pathogenic inflammation-coagulation cycle with harmful consequences for the placenta and developing fetus. In preeclampsia, an important non-infectious, life-threatening complication of pregnancy associated with significant maternal morbidity and poor birth outcomes, a role for the inflammation-coagulation cycle has been invoked [31]. Systemically elevated inflammatory cytokines and activated myeloid cells are features of this condition, as are profoundly dysregulated hemostasis and pathological placental fibrin deposition [31], [36]. Thus, PM shares important pathological features with clinical conditions known to involve inflammatory responses that are inexorably linked to dysregulated hemostasis, with significant implications for patient outcome.

Because the study from which the tested samples were derived was not designed or powered to measure coagulation or poor birth outcomes associated with PM, the results do not reveal associations between indicators of dysregulated hemostasis and preterm delivery or fetal growth restriction-associated LBW. However, infants born to infected women with elevated placental fibrin deposition did have reduced mean birth weights relative to uninfected cases with low fibrin. No soluble coagulation parameters were associated with birthweight in multivariate analysis. The weakness in our design notwithstanding, evidence of suppressed fibrinolysis (PAI-1 levels) did track with placental fibrin deposition, which has been shown in other studies to predict LBW [3], [6]; thus, future prospective studies to examine these associations in more detail are justified. In this context, testing of hemostatic parameters, including functional measures of coagulation and regulatory function, in the peripheral blood of malaria-exposed pregnant women will be critical, since detection of dysregulated hemostasis, if it is to be a potential therapeutic target, must be possible antenatally in venous blood. Because coagulation and fibrinolysis are significantly perturbed in severe *P. falciparum* malaria [42]–[47], providing compelling evidence for a pathogenic role of dysregulated hemostasis in PM, as in cerebral malaria [28], [48], will require coupling of functional coagulation metrics with identification of specific pathological outcomes, such as placental fibrin deposition, associated placental damage, premature birth and/or fetal growth restriction.

Investigation of profound pregnancy complications due to malaria, such as fetal loss, are difficult to conduct with human subjects due to ethical considerations, making the availability of mouse models very important. We have shown that *P. chabaudi* AS infection of B6 mice results in complete loss of pregnancy midgestationally [10], [11]; this corresponds to the time during which risk for malaria is highest in pregnant women (second trimester) and failure to protect against infection is associated with fetal loss [2], [49]. Importantly, antibody neutralization of TNF in IP B6 mice both restored midgestational pregnancy success and facilitated maintenance of placental TF expression at normal (low) levels [12]. The results herein confirm dysregulated hemostasis at the level of the conceptus in malaria-infected mice, with several indicators of activation and control of coagulation, as well as suppressors of coagulation and fibrinolysis, being increased at the level of gene expression. Likewise, mRNA for PARs 1 and 2 are upregulated. The results also provide evidence that in addition to maternal monocytic promotion of placental coagulation through upregulation of TF [9], [12], the fetal trophoblast in contact with maternal blood may also help to tip the balance toward net increased production of fibrin with sustained, enhanced expression of TF [12] while TFPI and thrombomodulin decline. Cumulatively, the results suggest that, as in human PM, malarial infection in mice promotes a pathogenic inflammation-coagulation cycle with significant negative consequences for pregnancy.

The striking efficacy of LMWH treatment in restoring midgestation embryo viability in *P. chabaudi* AS-infected mice provides indirect evidence that coagulation plays a pivotal role in malaria-associated pregnancy loss. However, inflammatory responses are also operational in pregnancy loss in this model [12]. Thus, the independent pathogenic effects of inflammation and coagulation on placental and embryonic viability should be assessed, although the interconnectedness of the inflammation-coagulation cycle may make such dissection difficult. Ultimately, confirmation of a critical role for either pathway in malaria-induced compromise of pregnancy will still leave the molecular mechanisms that drive embryo loss in mice and fetoplacental damage in humans to be elucidated. Of particular interest for malaria-associated placental fibrin formation and fibrinolysis, it was recently shown that fibrin degradation products directly damage placental architecture via trophoblast cell death [50], [51]. Additionally, the role of PARs in the inflammation-coagulation cycle also should be considered. In a mouse model of bacterial sepsis and in human endotoxin challenge studies, interruption of coagulation in the absence of uncoupling of the inflammation-coagulation cycle, in which PARs are central, did not abrogate disease [52], [53]. Relevant to pregnancy specifically, recent evidence suggests that both PAR1 and 2 are important players in the pathogenesis of preeclampsia [54], [55].

A second more intriguing implication of LMWH-mediated rescue of pregnancy in malaria-infected mice is the potential for a novel therapeutic intervention based on anticoagulant treatment for pregnancies at risk for malaria-associated poor birth outcomes. Because even a submicroscopic level of placental infection is associated with dysregulated hemostasis and a short-lived, rapidly treated infection during pregnancy can still have an adverse impact on birth outcome [56], [57], it is plausible that the coagulation-inflammation cycle continues to cause coagulopathy in the placenta even after curative antimalarial treatment is delivered. Therefore, simultaneous disruption of coagulation (and therefore coagulation protease-driven inflammatory responses through PARs) and removal of inflammation-inducing iRBCs with combination antimalarial/anticoagulant drug treatments may be more effective in preventing PM-induced pre-term labor and LBW. There is historic precedent for this approach in treatment of severe malaria in non-pregnant patients. Two early studies showed that treatment of pediatric cerebral malaria with unfractionated heparin and antimalarial drug reduced morbidity and mortality [58], [59]. Despite this success, however, failure of heparin therapy in two of three trials in rhesus macaques [60]–[62], and concerns about severe bleeding precipitating patient death in association with unfractionated heparin use [63]–[65] have compelled the World Health Organization to concur with concerned scientists that anticoagulant treatment should not be used in malaria therapy [60], [66]–[68]. It is becoming increasingly clear, however, that the low molecular weight fractions of heparin (LMWH) retain excellent anticoagulant function but with greatly minimized bleeding-associated complications in treated patients [69]. Even within LMWHs, different manufacturing processes yield different structural fractions, yielding drugs with distinct activities and specificities that cannot be used interchangeably [70]. This may explain why a disparity in pregnancy success between mice treated with different LMWHs was observed; enoxaparin is generated by benzylation followed by alkaline hydrolysis, whereas the research grade LMWH used in this study was generated by oxidative depolymerization with Cu^2+^ and hydrogen peroxide, which is the method used to create the LMWH, parnaparin [71].

Aside from inhibition of coagulation, glycoconjugates, including fractions of heparin, have potential adjunctive therapeutic value for severe malaria syndromes due to activity in iRBC rosette disruption, blockage of merozoite invasion and inhibition of iRBC sequestration [72]–[76]. Such glycoconjugates have little to no anticoagulant activity, yet in at least one case, some clinical benefit was observed following administration of curdlan sulfate in severe/cerebral malaria patients [77]. The paucity of contemporary efforts to test the efficacy of anticoagulant treatment in severe malaria syndromes, such as PM, might therefore remain given fears of bleeding and the promise of other glycoconjugate-based adjuncts which act directly on the parasite and/or iRBC. Nonetheless, the data presented herein demonstrate that humans express markers for malaria-induced dysregulated hemostasis during PM and a rodent model of PM exhibits enhanced midgestational embryonic survival upon treatment with LMWH. Importantly, *P. chabaudi* is known to form rosettes, but these rosettes, unlike those of *P. falciparum*, are insensitive to glycoconjugate treatment [78]. As reported here, infection kinetics were not different in LMWH-treated and -untreated IP mice. Thus, the improvement in midgestational status of treated mice suggests that dysregulated hemostasis leading to a procoagulant environment is at least partially responsible for malaria-induced embryo loss, and suppression of coagulation protects against this outcome.

Further work to demonstrate the efficacy of anticoagulant therapy to allow murine pregnancies to proceed to term, reverse coagulopathy already established in the placenta, and improve outcomes in concert with anti-malarial treatment remains to be achieved. In the meantime, however, the present results warrant prospective, longitudinal investigations in malaria-exposed women to establish the presence, antenatally, of dysregulated hemostasis in association with infection, and identify the extent to which this hemostatic disruption predicts placental coagulopathy and poor birth outcomes. Should clear associations be found and confirmations in rodent models be achieved, then evaluation of the safety and efficacy of anticoagulants as an adjunctive treatment to antenatal, curative anti-malarial treatment may be considered. Importantly, hemostatic disorders in pregnancy are currently safely and successfully treated with such therapies [79]–[82]. Although first generation anticoagulant treatment for malaria met with clinical failure due to bleeding complications [63]–[65], this risk is much lower with the new generation drugs. The latest generation is available in oral formulations, which although not currently indicated for use in pregnant women, may with further development and safety testing make delivery and patient compliance more facile. Overall, while a small risk of bleeding complications remains with any anticoagulant treatment, and is especially relevant for parturient women who are at risk for peri- and post-partum hemorrhage, the potential benefits of limited, monitored inclusion of drugs like LMWH in treatment for malaria during pregnancy deserves careful consideration.

Recognition that pathogenesis in both PM [12] and cerebral malaria [28], [48], [83], [84] is mediated by the inflammation-coagulation cycle is likely to become increasingly relevant, particularly in the critical search for much-needed novel therapies. In our mouse model for PM, targeting either inflammation [12] or coagulation provides significant clinical benefit. A recent study by Francischetti and colleagues [83] showed that defibrotide, a nucleotide-based drug [85]–[87], has multipotent effects against malaria-induced cellular activation, inflammatory responses and dysregulated hemostasis, and delayed disease development in a murine model for CM. Interestingly, although defibrotide has low intrinsic anticoagulant activity, it effectively interferes with TF function, thrombin generation, and platelet activation [83], [85]–[87]. Common among all of these treatment strategies is interruption of the inflammation-coagulation cycle. Thus, further study of the molecular events at the intersection of this pathogenic cycle in model systems and affected human populations has the potential to reveal critical, novel targets in the host response to malaria that contribute substantially to pathogenesis.

## Supporting Information

Figure S1 **Stereological assessment of fibrin in placental sections correlates with semi-quantitative scoring method.** Photomicrographs of tissue sections at 200× final magnification were captured. One image each from the basal and chorionic plates and eleven randomly selected intervillous regions spanning the full thickness of the placental disk, each representing an area measuring 615 µm×460 µm (2.83×10^5^ µm^2^), were assessed. Using GNU Image Manipulation Program (v2.6), a grid of 30 µm×30 µm was superimposed over the images, and at each intersection on the grid (300 total per image) the structural component present was scored. Components scored were basal plate, chorionic plate, villus (stroma and trophoblast), fetal blood vessel, syncytial knot, intervillous space, and intervillous or perivillous fibrin deposition. Villi converted to fibrinoid-type fibrin were counted as fibrin deposition. Fibrin score, represented as a percentage of intervillous space occupied, was calculated using the following formula: (total number of grid intersections scored as fibrin/(fibrin intersections+intervillous space intersections))×100.
(TIF)
