# Supplementary figures and images for: Maternal Malaria Induces a Procoagulant and Antifibrinolytic State That Is Embryotoxic but Responsive to Anticoagulant Therapy
Source: PLoS One. 2012 Feb 7;7(2):e31090. doi: 10.1371/journal.pone.0031090 (PMC3274552; doi:10.1371/journal.pone.0031090)

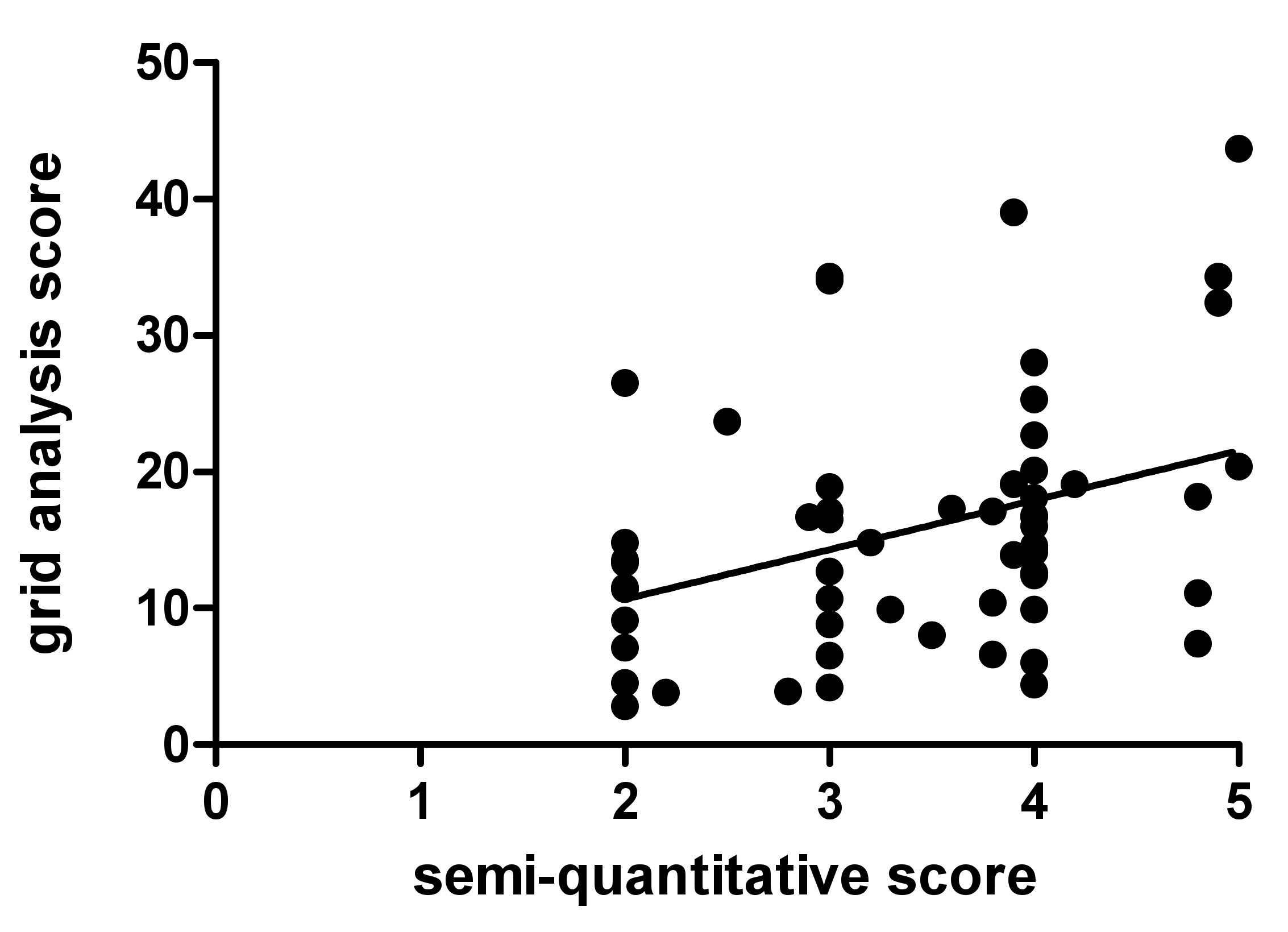

Supplement: Figure S1 — Stereological assessment of fibrin in placental sections correlates with semi-quantitative scoring method. Photomicrographs of tissue sections at 200× final magnification were captured. One image each from the basal and chorionic plates and eleven randomly selected intervillous regions spanning the full thickness of the placental disk, each representing an area measuring 615 µm×460 µm (2.83×10^5^ µm2), were assessed. Using GNU Image Manipulation Program (v2.6), a grid of 30 µm×30 µm was superimposed over the images, and at each intersection on the grid (300 total per image) the structural component present was scored. Components scored were basal plate, chorionic plate, villus (stroma and trophoblast), fetal blood vessel, syncytial knot, intervillous space, and intervillous or perivillous fibrin deposition. Villi converted to fibrinoid-type fibrin were counted as fibrin deposition. Fibrin score, represented as a percentage of intervillous space occupied, was calculated using the following formula: (total number of grid intersections scored as fibrin/(fibrin intersections+intervillous space intersections))×100. (TIF) [file pone.0031090.s001.tif]
